# Phylogeographic Patterns of Haemoproteid Assemblages of Selected Avian Hosts: Ecological and Evolutionary Implications

**DOI:** 10.3390/microorganisms10051019

**Published:** 2022-05-12

**Authors:** Alžbeta Šujanová, Radovan Václav

**Affiliations:** Institute of Zoology, Slovak Academy of Sciences, Dúbravská Cesta 9, 84506 Bratislava, Slovakia; alzbeta.sujanova@savba.sk

**Keywords:** haemoproteid parasites, avian hosts, climate, Eurasian blackcap, Western Palearctic

## Abstract

Background: While the dynamics of disease emergence is driven by host–parasite interactions, the structure and dynamics of these interactions are still poorly understood. Here we study the phylogenetic and morphological clustering of haemosporidian parasite lineages in a local avian host community. Subsequently, we examine geographical patterns of parasite assemblages in selected avian hosts breeding in Europe. Methods: We conduct phylogenetic and haplotype network analyses of *Haemoproteus* (*Parahaemoproteus*) lineages based on a short and an extended cytochrome *b* barcode region. Ordination analyses are used to examine changes in parasite assemblages with respect to climate type and geography. Results: We reveal relatively low phylogenetic clustering of haemoproteid lineages in a local avian host community and identify a potentially new *Haemoproteus* morphospecies. Further, we find that climate is effectively capturing geographical changes in parasite assemblages in selected widespread avian hosts. Moreover, parasite assemblages are found to vary distinctly across the host’s breeding range, even within a single avian host. Conclusions: This study suggests that a few keystone hosts can be important for the local phylogenetic and morphological clustering of haemoproteid parasites. Host spatio-temporal dynamics, both for partially and long-distance migratory birds, appear to explain geographical variation in haemoproteid parasite assemblages. This study also gives support to the idea that climate variation in terms of rainfall seasonality can be linked to the propensity for host switching in haemosporidians.

## 1. Introduction

Most of the emerging diseases are caused by zoonotic pathogens, but the spatio-temporal patterns of their emergence are not clearly understood [1]. While it is accepted that the dynamics of disease emergence is driven by host–parasite interactions [2], the structure and dynamics of these interactions is known to be complex [3,4]. One of the key questions in disease ecology, therefore, is what are the overarching factors driving the structure of parasite assemblages [5]. In this respect, avian haemosporidians represent an excellent study system due to their high diversity and potential to spread among hosts over vast geographical areas [6,7,8].

Morphological and molecular phylogenies of animal hosts and their parasites generally show parallel cladogenesis, pointing to an important role of shared evolutionary histories [9]. Indeed, the evolution and distribution of avian haemosporidians are fairly conservative with respect to host phylogeny and geography, although host sharing and host switching occur frequently at shallow depths within the parasite phylogenetic tree [3,4,8]. Additionally, inferring shared evolutionary histories between parasites and their avian hosts is challenging due to diverse host migratory strategies complicating the phylogeographic patterns of host–parasite associations [10]. Nonetheless, results yielded for certain ecological systems, such as archipelagos, demonstrate that host–parasite evolution histories routinely involve repeated events of co-divergence as well as host switching and colonizations [11,12]. The ubiquity of host switching and colonization has stimulated multiple studies that attempted to explore the roles of ecological conditions for these processes [5]. Importantly, host-related ecological traits of parasite infections, such as variation in avian host immune responses [13], body size [14], or habitat associations [15], are often phylogenetically conserved (see [16] for avian immune responses). Unsurprisingly, phylogenetic and ecological effects on various parasite traits such as prevalence, abundance, or host specificity are difficult to disentangle at broader geographical and host ranges [5,17].

Avian life-history strategy is an important ecological trait known to covary with haemosporidian prevalences [5,15,17,18]. In general, parasites that infect long-distance migratory birds show a broader geographical distribution and diversity, and these bird species are exposed to a higher abundance and diversity of vectors [19]. Indeed, migration distance was found to be positively linked to the probability of haematozoa infections in some bird species [20]. However, a recent comparative study has only corroborated this link for *Leucocytozoon* parasites [17]. In turn, infection probability for parasites of the subgenera *Parahaemoproteus* was found to increase with bird body mass and species richness, canopy-foraging type, and forest cover [17]. Even so, the pattern of infection probability was not consistent across different zoogeographical zones, in some cases even showing a reversed pattern, suggesting that important ecological processes behind infections take place within avian communities at regional spatial scales [17]. The latter assertion is important because data on host–parasite associations mostly come from locally-restricted studies focusing on individual avian host species or parasite lineages (e.g., [21,22,23,24,25,26,27,28]). 

Understanding the drivers of host–haemosporidian associations is also difficult due to numerous methodological challenges. For example, while phylogenetic analysis based on a short cytochrome *b* (*cytb*) fragment of the mitochondrial DNA (mtDNA) implies a monophyletic origin of the subgenera *Haemoproteus* and *Parahaemoproteus* [27], a phylogenetic hypothesis based on the complete mitochondrial genome suggests a paraphyletic arrangements of the two subgenera [29]. To address this problem, in addition to examining longer DNA sequences and employing different molecular markers [30], haplotype network analysis has been used to aid in understanding the associations between avian host taxa, insect vectors, and parasite molecular and morphological characteristics. With this approach, for example, it was possible to identify for the haemosporidian parasites of thrushes (Passeriformes: Turdidae) the molecular lineages of evolutionary importance, which had not previously been described morphologically [28,31]. Such findings are important because the same morphological species can constitute a complex of cryptic lineages with different fitness effects on their hosts and/or vectors [32], while even genetically closely related lineages, particularly if based on mtDNA, can represent morphologically and biologically distinct species [31].

Here we build on a previous study by Šujanová et al. [33], where diversity, host specificity, prevalence, and seasonality of haemosporidian parasites was examined for a local avian community in Slovakia. That study revealed pronounced seasonality in the abundance of haemosporidian *cytb* lineages, while only a subset of all the lineages, mainly comprising those of *Haemoproteus* parasites, was detected in locally breeding avian hosts. In this study we conduct phylogenetic and haplotype network analyses for *Haemoproteus cytb* lineages to elucidate the phylogenetic structure of parasite lineages and morphospecies detected in the local avian community. Furthermore, we analyze the presence and abundance of *Haemoproteus* lineages by combining our data with that from the MalAvi database [34] to reveal geographical variation in parasite assemblages in selected avian hosts breeding in Europe.

## 2. Materials and Methods

### 2.1. Study Area, Field Methods and Study Species

**Figure 1:**
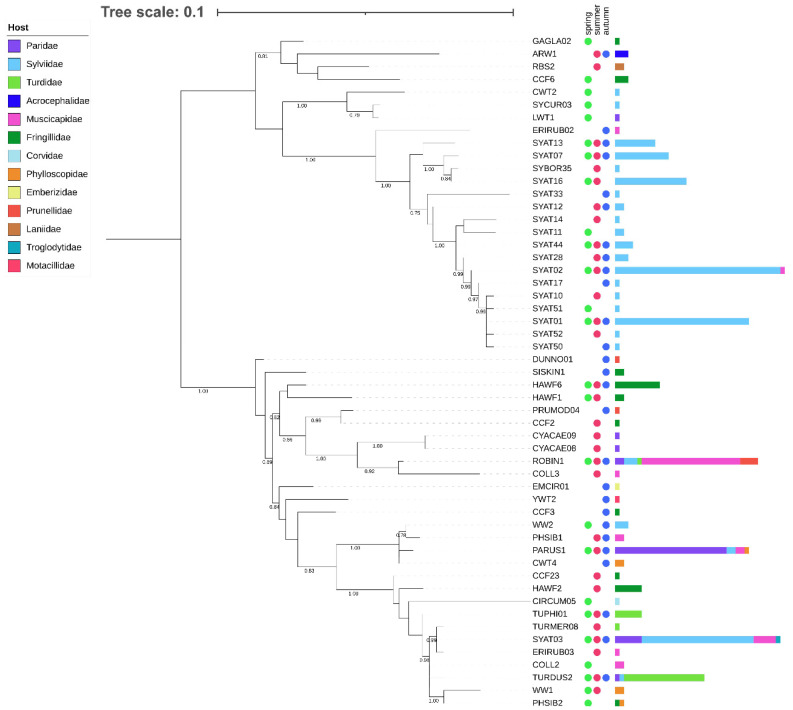
Maximum likelihood tree of avian haemoproteid lineages detected at the study site in Slovakia based on a 478 bp mtDNA region of the *cytb* gene. The tree was constructed based on the best-fit substitution model (TIM2 + F + I + G4) and is rooted at midpoint. The branch support was assessed using the aBayes test, and the corresponding values are shown for branches >75% support. After each lineage, circles denote the seasonal dynamics of the lineage’s occurrence and horizontal bars denote the lineage’s abundance in their avian hosts of 13 families.

Wild birds were sampled in Slovakia at the Drienovec Bird Ringing Station (48°36′58.7″ N; 20°54′53.6″ E) as a part of research on vector-borne avian pathogens [33,35]. The study site (ca. 7.7 ha) is represented by a mosaic of woody wetland and forest meadow ecotones at 190 m a.s.l. Birds were captured, banded, and sampled under the permits of the Ministry of the Environment of the Slovak Republic No. 269/132/05-5.1_p and 9830/2017-6.3. Birds were captured using mist nets over a period of three years (2017, 2018, 2019). The blood sample was taken from a brachial vein and ring code, species, and, if possible, age and sex were recorded for each bird sampled, and the birds were subsequently released. Blood samples were stored in 70% ethanol at 4 °C until DNA extraction (within 7 months). In total, 1851 birds of 61 species were blood-sampled (spring: April, *n* = 444; summer: June–July, *n* = 474; autumn: September–November, *n* = 933; Figure 1). Birds were sampled each year in the second half of April, between mid-June and mid-July, and between mid-September and the beginning of November. These three sampling periods were chosen for our study area and climatic zone to obtain representative samples for haemosporidian assemblage composition in birds during spring migration, breeding, and autumn migration periods, respectively. In this study, we examine data for 300 *Haemoproteus*-positive samples with known *cytb* lineage identity that were detected in 26 passerines by Šujanová et al. [33].

### 2.2. DNA Extraction

DNA extractions were performed using a QIAamp DNA Blood Kit (Qiagen, Hilden, Germany), following the manufacturer’s recommendation. Extracted DNA was resolved to a final concentration of ca. 100 ng/μL and stored at −20 °C until subsequent analyses. The quantity and quality of DNA samples was assessed by NanoPhotometer Pearl (Implen, Munich, Germany).

### 2.3. PCR Analyses

DNA samples were analyzed by two PCR protocols. First, the nested PCR assay targeted a mtDNA *cytb* gene fragment [36]. In the first step, HaemNFI and HaemNR3 primers were used to amplify the 617 bp fragment. PCR reactions were performed in 20 cycles, which included initial denaturation at 94 °C for 2 min, followed by 20 cycles of denaturation at 94 °C for 30 s, annealing at 50 °C for 30 s, elongation at 72 °C for 45 s, and consequently final elongation at 72 °C for 10 min. Subsequently, the amplicon was added as a template to the second step of nested PCR with primers HaemF and HaemR2, targeting a 480 bp amplicon of *Haemoproteus* sp. The PCR protocol was adjusted so that the number of cycles was raised to 35. Each reaction with the total volume of 10 μL contained 1 μL of template DNA (ca. 20 ng), 5 μL of SuperHot Master Mix (2×) (Bioron, Ludwigshafen, Germany), 0.25 μL of each primer (with concentrations 10 pmol/μL), and 3.25 μL of miliQ water and 0.25 μL MgCl_2_.

The second PCR assay targeted a 1773 bp sequence of mtDNA encompassing the *cytb* gene fragment [37]. PCR reactions were performed with primers AE298-EF and AE299-ER in 35 cycles, which included initial denaturation at 94 °C for 2 min, followed by 35 cycles of denaturation at 94 °C for 45 s, annealing at 54 °C for 45 s, elongation at 72 °C for 60 s, and consequently final elongation at 72 °C for 10 min. Each reaction with the total volume of 20 μL contained 3 μL of template DNA (ca. 20 ng), 10 μL of SuperHot Master Mix (2×) (Bioron, Ludwigshafen, Germany), 0.5 μL of each primer (with concentrations 10 pmol/μL), and 5.5 μL of miliQ water and 0.5 μL MgCl_2_.

PCR products from both PCR assays were separated and visualized on 2% agarose gel with SYBR^®^ Safe DNA gel stain (Invitrogen, Carlsbad, CA, USA). Positive PCR products were purified using a Qiagen purification kit (Qiagen, Hilden, Germany). The purified PCR fragments were sent for sequencing in both directions (Macrogen Europe, Amsterdam, The Netherlands).

The sequences were edited, aligned, and trimmed to the same length using Unipro UGENE software v1.32 [38]. The resulting contigs of 478 and 1516 bp were examined with the BLASTn algorithm in GenBank (http://www.blast.ncbi.nlm.nih.gov/Blast.cgi (accessed on 1 February 2022)) and MalAvi [34] (http://130.235.244.92/Malavi/blast.html (accessed on 1 February 2022)) databases. All (24) unique 1516-bp nucleotide sequences were deposited in GenBank: ON138422–ON138444 (*Haemoproteus* sp. lineages ROBIN1, LWT1, SYAT03, TURDUS2, YWT2, RBS2, SYAT10, PARUS1, CCF2, COLL3, SYAT01, HAWF2, TUPHI01, SYAT02, WW2, DUNNO01, EMCIR01, COLL2, SISKIN1, SYAT16, SYAT52, SYAT11, and PHSIB2) and ON146446 (*Haemoproteus* sp. lineage CCF6). The 478-bp sequences used in this study are as reported previously by Šujanová et al. [33].

### 2.4. Phylogenetic and Haplotype Network Analyses

We estimated maximum likelihood phylogenies for 53 lineages with 478-bp nucleotide sequences (short *cytb* barcode region) and 24 lineages with 1516-bp nucleotide sequences (extended *cytb* barcode region) using the IQ-TREE web application [39]. The best-fit substitution model of sequence evolution was selected using the ModelFinder within the IQ-TREE platform [40]. The ModelFinder considers all traditional substitution models included in jModelTest and ProtTest [41,42], but also includes discrete Gamma (+G) [43] and FreeRate (+R) heterogeneity [44] models, the latter representing a generalization of the discrete Gamma model. The best-fit model was selected with respect to Bayesian Information Criterion (BIC) scores. The same substitution model, TIM2 + F + I + G4, was selected as the best-fit model for both phylogenies. The best-fit models were clearly superior to the second-best-fit models (BIC weights for the best-fit vs. second-best-fit models: 87.8% vs. 11.9% for the short-barcode tree; 97.9% vs. 1.4% for the extended-barcode tree). The branch support for the tree with the best substitution model was assessed using the aBayes test [45]. The resulting trees for both phylogenies were rooted at midpoint, because this is a preferred option when optimal outgroups are not available [46], as was our case with the tree based on 1516 bp sequences. The trees were edited in iTOL v5 [47].

The same two sets of sequences as used for phylogenetic analyses were used for the haplotype networks analysis. Haplotype networks were constructed using the median-joining network algorithm [48] with PopART 1.7 [49]. 

### 2.5. Statistical Analyses

Ordination analyses were used to examine the importance of the environmental factors involved in geographical changes in haemoproteid assemblages, focusing on the parasite lineages of avian hosts relevant for our study site in Slovakia. First, we used constrained canonical analysis (CCA) to examine whether the changes in haemoproteid assemblages across the Western Palearctic can be captured by latitude and/or climate as proxies of environmental variation [50]. For this analysis, we compiled data from the MalAvi database [34] (http://130.235.244.92/Malavi/ (accessed on 1 February 2022)) on lineage *presence* at individual study sites for five avian species, which are widespread in Europe while also representing common haemoproteid hosts: Eurasian blackcap *Sylvia atricapilla*, Eurasian blackbird *Turdus merula*, great tit *Parus major*, common chaffinch *Fringilla coelebs*, and Eurasian blue tit *Cyanistes caeruleus*. In total, we compiled data on the presence of 71 haemoproteid lineages for 68 sites from 21 countries, including the data for our study site (Tables S1 and S3). Climate type and latitude were used as constraining environmental variables, whereas climate was classified according to the Köppen–Geiger classification system [51]. Specifically, we assigned all study sites to one of four climate types: Mediterranean (subtypes: *Csa*—dry season and hot summer, *Csb*—dry season and warm summer, and *BSk*—dry season and warm to hot summer), warm temperate (*Cfa*—no dry season and hot summer), temperate oceanic (*Cfb*—no dry season and warm summer), and humid continental (*Dfb*—no dry season and warm summer). This climate categorization was selected because it succinctly describes climate variation in the Western Palearctic while also capturing geographical variation in the parasite data used. The CCA biplot was constructed given the scaling 1, whereby individual objects (haemoproteid lineages) found near the centroid for a categorical explanatory variable (climate) are more likely to show presence for such climate type, and the distances between individual objects and centroids approximate χ^2^ distances [52]. A forward model selection based on the maximization of adjusted *R*^2^ values was used to obtain the most parsimonious explanation of our data [53].

Second, we used principal component analysis (PCA) to examine the structure of haemoproteid assemblages of blackcaps at six study sites across Europe, including our study site. We compiled data from the MalAvi database (accessed on 1 February 2022) for six study sites with rigorous data on the *abundance* of 39 haemoproteid lineages detected in blackcaps (Tables S2 and S4). The haemoproteid abundance data was Hellinger-transformed before analysis to reduce the importance of large lineage abundance values and double zeros [52]. The PCA biplot was constructed given the scaling 1 (distance plot), which serves to show site clustering and the position of descriptors (haemoproteid lineages) with respect to sites [52]. Both ordination analyses were conducted with the *vegan* package 2.5-7 [54] in the *R* software platform [55].

## 3. Results

### 3.1. Phylogenetic Analysis

**Figure 2:**
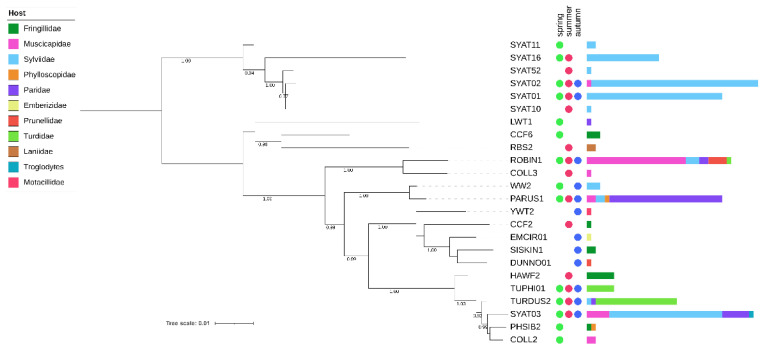
Maximum likelihood tree of avian haemoproteid lineages detected at the study site in Slovakia based on a 1516 bp mtDNA region encompassing the *cytb* gene. The tree was constructed based on the best-fit substitution model (TIM2 + F + I + G4) and is rooted at midpoint. The branch support was assessed using the aBayes test, and the corresponding values are shown for branches >75% support. After each lineage, circles denote the seasonal dynamics of the lineage’s occurrence and horizontal bars denote the lineage’s abundance in their avian hosts of 11 families.

We examined phylogenetic relationships for a local assemblage of haemoproteid lineages based on short (478 bp) and extended (1516 bp) *cytb* barcode regions (Figure 1 and Figure 2).

Phylogenies based on both barcode regions reveal that the local assemblage of haemoproteids comprises two major clades (Figure 1 and Figure 2). Clade 1 includes lineages related to *H. parabelopolskyi* and, with the exception of SYAT02 and ERIRUB02, they are associated with a single host species, the Eurasian blackcap *Sylvia atricapilla*. Clade 2 includes phylogenetically more diverse lineages, with each of the most frequently detected lineages (SYAT03, ROBIN1, PARUS1, and TURDUS2) being associated with different host species of families Sylviidae, Muscicapidae, Paridae, and Turdidae.

### 3.2. Haplotype Network Analysis

**Figure 3:**
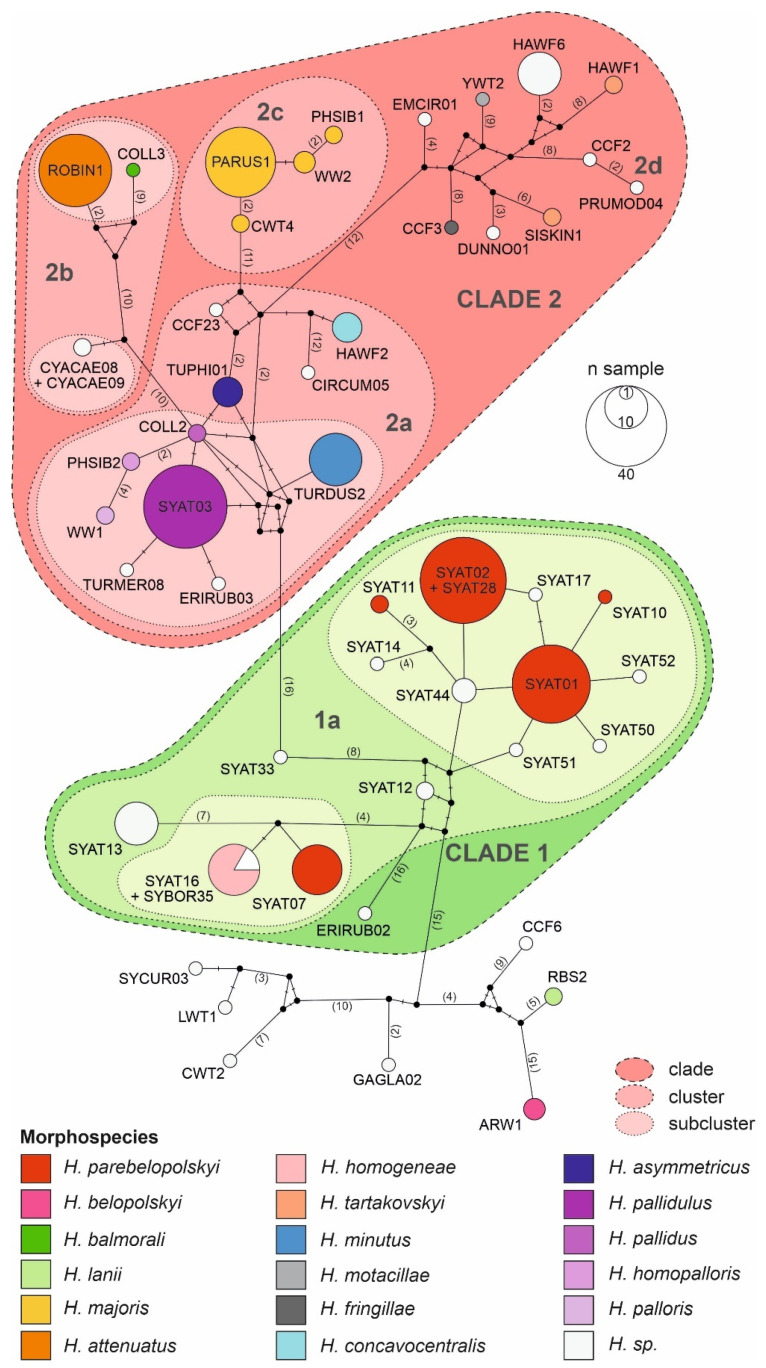
Haplotype network of avian haemoproteid lineages detected at the study site in Slovakia based on a 478 bp mtDNA region of the *cytb* gene. The haplotype network was constructed using the median-joining algorithm. The borders of lineage clades, clusters, and subclusters were delineated based on phylogenetic analysis. Haemoproteid morphospecies are shown for the corresponding lineages.

**Figure 4:**
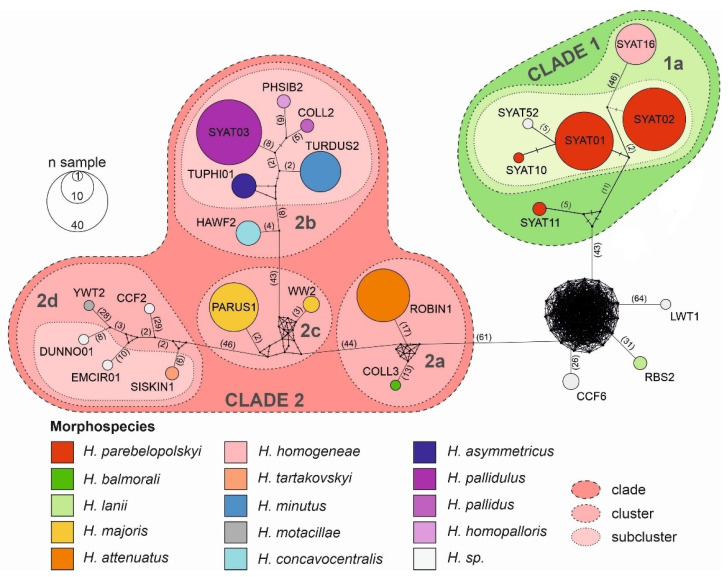
Haplotype network of avian haemoproteid lineages detected at the study site in Slovakia based on a 1516 bp mtDNA region encompassing the *cytb* gene. The haplotype network was constructed using the median-joining algorithm. The borders of lineage clades, clusters, and subclusters were delineated based on phylogenetic analysis. Haemoproteid morphospecies are shown for the corresponding lineages.

Results of haplotype network and phylogenetic analyses reveal that the local assemblage of haemoproteids can be grouped into five major morphospecies clusters (Figure 3 and Figure 4).

The cluster including most frequently locally detected lineages, which are related to *H. parabelopolskyi*, corresponds to clade 1 (Figure 3 and Figure 4). The second dominant morphospecies cluster (2a) comprises lineages belonging to *H. pallidulus*, *H. pallidus*, *H. homopalloris*, *H. palloris*, *H. minutus*, and *H. concavocentralis*. The lineages TURMER08 and ERIRUB03, which were only detected at our study site, are most closely related to *H. pallidulus* (Figure 1 and Figure 3). The third morphospecies cluster (2b) comprises lineages related to *H. balmorali* as well as two distinct, but morphologically undescribed, lineages CYACAE08–09. The fourth morphospecies cluster (2c) comprises lineages belonging to a single morphospecies *H. majoris* (Figure 3 and Figure 4). Finally, the fifth morphospecies cluster (2d), based on the extended barcode region, comprises lineages belonging to *H. tartakovskyi* and *H. motacillae* as well as morphologically undescribed lineages DUNNO01, EMCIR01, and CCF2.

### 3.3. Ordination Analyses

**Figure 5:**
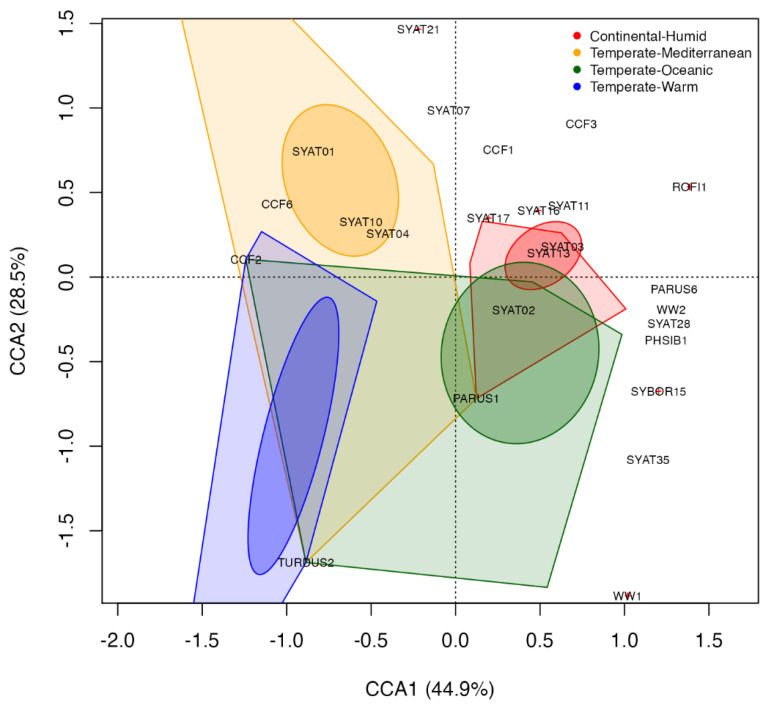
Constrained canonical analysis biplot of haemoproteid presence data for five widespread European avian hosts at 68 sites in the Western Palearctic. Haemoproteid presence data is constrained by a categorical environmental variable—climate type. The scaling 1 biplot shows ellipses representing 95% confidence intervals around each climate’s centroid and polygons representing the area created by connecting the outermost site scores for each climate. Red crosses show locations with overlapping lineages, in which cases the relatively most abundant lineage is shown (see Table S3 for CCA lineage scores).

First, we examine whether changes in the assemblages of *Haemoproteus* (*Parahaemoproteus*) lineages detected in different localities within the Western Palearctic in five widespread avian host species can be captured by latitude and/or climate. Based on a model building approach by maximization of adjusted-*R*^2^, the parasite assemblage is most parsimoniously captured by climate (model’s adjusted-*R*^2^: climate model = 3.3%, latitude model = 1.5%). Climate explained small, but significant, unadjusted-proportion (8%) of inertia in the parasite occurrence data (constrained canonical analysis, CCA: χ^2^ = 0.84, df = 3, *p* < 0.01). Only the first CCA component explains a significant proportion of inertia (CCA1: χ^2^ = 0.37, df = 1, *p* = 0.032; CCA2: χ^2^ = 0.24, df = 1, *p* = 0.20). As for the CCA results, the Mediterranean climate captures the occurrence of lineages SYAT01, SYAT10, SYAT04, and CCF6, while the occurrence of lineage TURDUS2 is most efficiently explained by the warm temperate climate (Figure 5 and Table S3).

With respect to climates of higher latitudes, the temperate oceanic climate is effectively explaining the occurrence of SYAT02 and PARUS1, while lineages SYAT03, SYAT11, SYAT13–14, and SYAT16 are typical for the humid continental climate (Figure 5 and Table S3).

**Figure 6:**
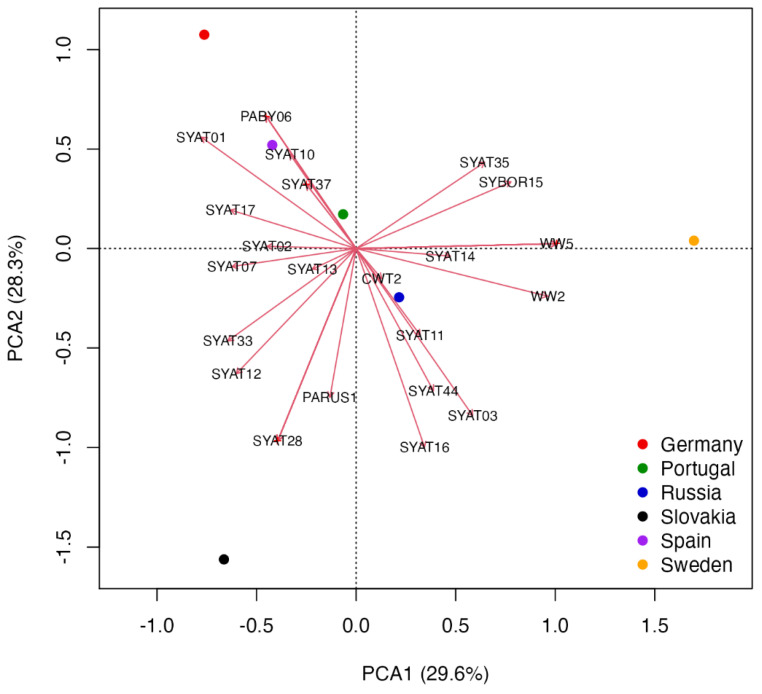
Principal component analysis biplot of haemoproteid abundance data at six study sites in Europe. The scaling 1 biplot shows centroids representing site scores and arrows representing haemoproteid abundance scores. Red crosses show locations with overlapping lineages, in which cases the relatively most abundant lineage is shown (see Table S4 for PCA lineage scores).

Further, we use principal component analysis (PCA) to examine how assemblages of haemoproteid lineages detected in *Sylvia atricapilla* vary at six different localities within Europe (Figure 6).

The first two PCAs represent the most important components according to the broken stick method. The analysis reveals that blackcaps sampled in German, Spanish, and Portuguese sites show a relatively highest abundances of PABY06, SYAT01, SYAT04, SYAT10, SYAT30–32, SYAT34, and SYAT36–37, while the blackcaps from the same sites show a relatively lowest abundance of SYAT03, SYAT11, SYAT16, and SYAT44 (Figure 6 and Table S4). The analysis also reveals distinct geographical associations of blackcaps with lineages, which are not typical for this species. Namely, while blackcaps at the Slovak site show associations with PARUS1, ROBIN1, and TURDUS2, blackcaps sampled at the Swedish site show associations with CCF2, CWT4, WW2, and WW5 (Figure 6 and Table S4).

## 4. Discussion

This study was aimed at unravelling the phylogeographic patterns of haemoproteid assemblages in an avian host community in Slovakia as well as in selected avian hosts breeding in Europe. Our analyses, based on the *cytb* barcode region, reveal that locally occurring haemoproteid lineages constitute two major well-supported clades. It is noteworthy that most lineages of clade 1 are dominantly associated with a single avian host, the Eurasian blackcap *Sylvia atricapilla* (hereafter blackcap), and blackcaps are locally associated with haemoproteids of all major morphospecies clusters, including the cluster with pale-staining highly pathogenic haemoproteids related to *H. minutus*, stressing the importance of this warbler species for the local host–parasite network structure.

Our phylogenetic results for locally occurring haemoproteids are in general agreement with previous studies [23,27,28,31,56,57,58]. Most of the lineages, which have previously been detected throughout the breeding range of blackcaps, constitute clade 1. While these lineages show close relatedness to *H.* (*Parahaemoproteus*) *parabelopolskyi*, the clade represents a phylogenetically and ecologically heterogeneous group of blackcap haemoproteids [58,59]. The type specimens for *H. parabelopolskyi* correspond to the lineage SYAT02 [60], but multiple other related lineages (SYAT01, SYAT04, SYAT07, SYAT10, SYAT11, or SYAT16) have often been considered as intraspecific variation of this morphospecies [23,56,58]. Nonetheless, the lineage SYAT16 is morphologically and molecularly different from lineages SYAT01–02, and the former lineage is currently recognized as a distinct morphospecies, *H. homogeneae* [27]. Our phylogenetic and haplotype network analyses support this taxonomic change and suggest that the lineage SYAT11 may as well constitute a distinct morphospecies in this clade.

Of clade 2, the epidemiologically most important morphospecies cluster is represented by haemoproteids with pale-staining cytoplasm (cluster 2a), including a highly pathogenic *H. minutus* [18,27,61]. This cluster comprises locally abundant lineages of common avian taxa (family Sylviidae—SYAT03; Turdidae—TURDUS2-TUPHI01; Fringillidae—HAWF2). The summer occurrence of these lineages at our study site [33] is in accordance with previous studies implicating *Culicoides* biting midges as vectors for these lineages within Europe [57,62,63,64]. Based on the haplotype analysis of *Haemoproteus* lineages in thrushes (*Turdus* spp.), the lineage TUPHI1 was identified as a possible cryptic species of *H. minutus* [28]. Subsequent morphological analysis confirmed this suspicion, and the lineage has recently been described as *H. asymmetricus* [31]. Harl et al. [28] observed that the lineages TUPHI1 and TURDUS2 are not directly connected in the haplotype network, but via the intermediate haplotype COLL2 belonging to a morphologically distinct *H. pallidus*. Our results based on the extended barcode region differ from the latter work and suggest that the lineages COLL2, TUPHI1, and TURDUS2 form a paraphyletic and not a monophyletic group, thereby supporting the morphological insights obtained for these lineages. While the conventionally used short *cytb* barcode region has proven to be useful in phylogenetic and morphological analysis [31], our work suggests that improved inference can be achieved by examining an extended barcode region for such closely related taxa.

The cluster (2c) comprising the lineages of *H. majoris* represents locally abundant heamoproteids. These lineages are typical for northern geographical regions of Europe [3,56,65] and were recorded at our study site mainly during the autumn migration period [33]. The four known *H. majoris* lineages show greater host generalism compared to typical haemoproteids and are considered to form a cryptic species complex [26]. Previously, we found that even though *H. majoris* lineages PARUS1 and WW2 show significant phylogenetic host specificity (PARUS1—Paridae; WW2—Sylviidae), the lineages are detected in diverse hosts within the local avian community and show low structural host specificity [33]. In this study, the two lineages were identified by ordination analysis as region-specific blackcap parasites (Figure 6). Specifically, while the lineage PARUS1 was characteristic for blackcaps in eastern Slovakia, blackcaps in southern Sweden and the Russian part of the Curonian Spit in the Baltic Sea were characterized by the lineage WW2 [5,33,56]. Interestingly, the lineage WW2 was not detected in any non-parid host in Slovakia, whereas at the Swedish and Russian sites the lineage WW2 was readily detected in multiple parid species [5,33,56]. Therefore, it seems that while host jumps are common for the two *H. majoris* lineages at higher European latitudes, this only applies to the lineage PARUS1 for lower latitudes. In a recent study in Sweden, Huang et al. [66] found seasonal variation in infection patterns between *H. majoris* lineages PARUS1 and WW2, suggesting that the variation might be related to the migratory strategies of their main avian hosts: long-distance migratory warblers—WW2 vs. partially migratory tits—PARUS1. We agree with Nilsson et al. [26] that the *H. majoris* species complex is an excellent study system on host switches and suggest that it might be worthwhile to examine the infection patterns of different *H. majoris* lineages, both for avian hosts and insect vectors, across broader geographical scales.

We found that climate effectively captures geographical variation in the presence of several of 71 haemoproteid lineages detected in five widespread European passerines (Figure 5). Considering the climates of lower European latitudes, the Mediterranean climate is strongly associated with the blackcap’s *H. parabelopolskyi* lineages SYAT01, SYAT04, and SYAT10, while the blackbird’s *H. minutus* lineage TURDUS2 is associated with the warm temperate climate, which represents a transitional climate between Mediterranean and humid continental climates. The haemoproteids of higher latitudes have been found to be relatively less structured based on climate. Nonetheless, the temperate oceanic climate appears to capture the occurrence of the blackcap’s *H. parabelopolskyi* lineage SYAT02 and the *H. majoris* lineage PARUS1, the latter lineage being typical for European parids. In turn, the humid continental climate appears to associate with the blackcap’s *H. pallidulus* lineage SYAT03, *H. homogeneae* lineage SYAT16, and the lineages SYAT11 and SYAT13–14. Recently, Harl et al. [28] examined geographical and avian host ranges for haemosporidians of thrushes (Turdidae), including the haemoproteid lineages TURDUS2, PARUS1, and SYAT03. The authors found that TURDUS2 is found mainly in Western Asia, PARUS1 in Eastern Europe, and SYAT03 in Western Europe. Even though our results are in apparent discordance, it is important to note that Harl et al. [28] classified geographical regions on a coarse spatial scale, e.g., western, southern, and northern Europe was treated as Western Europe. In fact, our results on TURDUS2 agree with [28] in that most of the lineage’s sites for Western Asia (sites in Armenia and the Krasnodar region in Russia) correspond to the region with the warm temperate climate. Our work therefore suggests that climatic and biogeographic variation can play an important role in parasite distribution at smaller geographical scales than currently appreciated.

In accordance with the assumption that the degree of niche specialization is a function of environmental heterogeneity [67], Fecchio et al. [68] found that local host specificity in avian *Plasmodium* and *Haemoproteus* parasites varies with climate in terms of rainfall seasonality. Interestingly, the relationship detected by Fecchio et al. [68] ran counter to the expectation based on environmental heterogeneity, revealing that the degree of local (realized) host specificity was higher for climates with greater rainfall seasonality. Our results, though based on a different set of haemosporidian lineages and climatic conditions, support the findings by Fecchio et al. [68]. Specifically, *H. parabelopolskyi* lineages SYAT01, SYAT04, and SYAT10, which show the affinity with the only of the four climates with rainfall seasonality, i.e., the Mediterranean climate, represent the lineages with the relatively greatest host specificity, both in terms of phylogenetic and realized host specificity [5,27,33,58,69,70]. Though the link between host specificity and precipitation heterogeneity is unclear, Fecchio et al. [68] suggested that higher spatial host concentration and infection prevalence in seasonal environments may favor host specialization over host jumps for both parasites and their blood-feeding vectors. Indeed, the year-round transmission strategy of the lineages SYAT01 and SYAT10 found for Spain [58] may facilitate the evolutionary response of these lineages towards host specialization, thereby stressing the perplexing roles of climate variation in pathogen emergence.

Our results, using the quantitative data for haemoproteids of blackcaps from six intensively sampled avian communities in Europe, provide a comparable picture to that based on the qualitative ordination analysis (Figure 5 and Figure 6). Specifically, the quantitative ordination analysis reveals that the lineages SYAT01 and SYAT10 are relatively most abundant in Portugal, Spain, and Germany, whereas the lineages SYAT03 and SYAT16 represent the relatively least abundant haemoproteids for the three countries. Even though the Russian site (the Curonian spit in the Baltic Sea) appears to most accurately describe the relative abundance of SYAT03 and SYAT16, the geographical inference is not strong. Instead, the Russian site appears to reflect a geographically intermediate blackcap population spanning Sweden and Slovakia where both SYAT03 and SYAT16 are relatively abundant [5,33]. Moreover, a weaker correlation between the relative abundance of SYAT02 and Portugal-Spain-Germany centroids, despite the high prevalence of SYAT02 at the German site [69], accords with our results on the affinity of SYAT02 with the temperate oceanic rather than the Mediterranean climate. Combined, our results suggest that the most common haemoproteids of blackcaps can be divided into three major ecotypes: (1) *H. parabelopolskyi* lineages SYAT01, SYAT04, and SYAT10, associated with the Mediterranean climate, (2) *H. parabelopolskyi* lineage SYAT02, associated with the temperate oceanic climate, and (3) *H. pallidulus* SYAT03 and *H. homogeneae* SYAT16, associated with the humid continental climate.

A direct question resulting from our study is, what are the possible determinants and consequences of geographical and phylogenetic structuring of the haemoproteids studied? Avian migratory strategies are often implicated as a key source of variation in haemosporidian distribution and prevalence [5,15,17,18,19]. For the community of woodland birds at our study site in Slovakia, the most abundant haemoproteids are associated with the most abundant partially-migratory passerines of families Paridae, Turdidae, and Muscicapidae [33]. A notable exception is the blackcap, a Sylviid warbler, showing complex migratory strategies ranging from non-migratory to long-distance migratory populations [71]. Of the dominant haemoproteid lineages detected at the study site in partially migratory birds, one is closely associated with the temperate oceanic climate (*H. majoris* PARUS1), another with the humid continental climate (*H. attenuatus* ROBIN1), and the third with the warm temperate climate (*H. minutus* TURDUS2). These findings are consistent with the traditional geographical strongholds of great tits and blue tits, robins, and blackbirds, respectively [72]. While showing distinct geographical affinities, the three morphospecies, but also *H. parabelopolskyi*, are transmitted by abundant generalist vectors such as *Culicoides impunctatus* and *C. nubeculosus* [73,74,75]. These biting midges are blood-sucking parasites, common mainly in northern Europe, showing a broad range of vertebrate hosts and serving as competent vectors for diverse haemoproteid species [74,75,76]. Importantly, the three haemoproteid morphospecies appear to form cryptic species complexes: *H. majoris* complex [26], *H. minutus* complex [28], and *H. balmorali* complex [77] and are implicated in serious health risks in naturally occurring but mainly naïve avian hosts [77,78,79,80]. A better understanding of the spatial distribution and health impact of individual lineages is needed for these morphologically cryptic haemoproteids, particularly for avian populations on the range margins of these haemoproteids. This is relevant because environmental changes such as climate change or landscape urbanization have shifted the centers of gravity northwards in many birds in Europe [81]. For example, great tits and blue tits are shifting their breeding ranges towards northeastern Europe [82], whereas blackbirds undergo colonization of European urban areas, likewise in a north-easternly direction [83].

### Implications

This study adds to the understanding of the phylogeographic pattern of haemoproteids in blackcaps. We found that, with respect to climatic conditions, haemoproteids of blackcaps appear to form three ecotypes: Mediterranean, oceanic, and continental. It is important to note that the Mediterranean ecotype in our study corresponds to the whole Mediterranean basin, not only to its western part represented by the Iberian Peninsula and north-western Africa. For example, the lineage SYAT10, which is according to our study assigned to the Mediterranean ecotype, was also detected in the eastern Mediterranean in Turkey [84]. Nonetheless, data for key regions of the Mediterranean ecotype, particularly for Greece and Italy, but also data for the oceanic ecotype from the UK, are almost completely lacking for blackcap haemoproteids (but see [85] for Italy’s Sardinia and GenBank data for the UK). Despite these geographical knowledge gaps, our study suggests that haemoproteids of blackcaps can be divided into two major groups with respect to their phylogeny and geographical affinity: (1) a group of phylogenetically more recent haemoproteids represented mainly by *H. parabelopolskyi* lineages SYAT01–02 with ranges in western and southwestern Europe and (2) a group of phylogenetically older haemoproteids represented by *H. pallidulus* lineage SYAT03 and *H. homogeneae* lineage SYAT16 with ranges in eastern and northeastern Europe. These findings are apparently consistent with the population ecology of blackcaps. First, blackcaps breeding in Europe are thought to comprise eastern and western populations with respect to their migration routes [86]. Second, since the middle of the last century blackcaps show changes in migratory strategies westwards and the establishment of stable wintering populations in western and southwestern Europe [71]. It is possible that the evolution of *H. parabelopolskyi* lineages SYAT01–02 may be linked to these changes in blackcaps’ migratory behavior. Importantly, blackcaps are not restricted to overwintering only in western Europe. A substantial proportion of blackcap populations, including blackcaps breeding in Slovakia [87], is wintering in the Apennine Peninsula and Greece [88]. Even though the parasitological data is almost absent for these European regions, it is possible that the phylogenetically older group of blackcap haemoproteids is associated with blackcaps using these central and eastern European regions for wintering or as stopover sites. In fact, *Haemoproteus* lineage SYAT44, which is similarly as lineages SYAT03 and SYAT16 associated with blackcap populations in eastern and northeastern Europe (Figure 6), was detected in blackcaps in Sardinia [85]. This question deserves attention because the recent changes in genetically-based migratory strategies appear to have important fitness consequences for blackcaps [89]. Specifically, the link between greater reproductive success and advanced timing of breeding in blackcaps wintering in western Europe, as suggested by Bearhop et al. [89], should be treated with caution. First, given the relatedness of *H. pallidulus* SYAT03 to the highly pathogenic pale-staining haemoproteids including *H. minutus*, the haemoproteids of eastern and western populations of blackcaps can differ in their pathogenicity. Second, previous works revealed that haemoproteids associated with eastern populations of blackcaps (*H. pallidulus* SYAT03 and *H. homogeneae* SYAT16) show a spring–summer transmission strategy, whereas the haemoproteids associated with the blackcap’s western populations (*H. parabelopolskyi* SYAT01–02) show a summer–autumn transmission strategy [33,58,59]. Therefore, different effects on host health and condition of distinct blackcap haemoproteids can contribute to variation in the reproductive performance of blackcaps with different migratory strategies.

## 5. Conclusions

Our work emphasizes the need to improve the understanding of host determinants of geographical variation in the structure of parasite assemblages, because host spatio-temporal dynamics can have important consequences, not only for host infection patterns [19], but also for parasite evolutionary responses [90,91]. Moreover, given the environmentally-driven shifts in the distribution of keystone haemoproteid hosts with broad ranges such as great tits, blackbirds or blackcaps, the phylogeographic patterns of host–parasite associations can have important consequences for pathogen emergence in naïve host populations [92]. Avian haemosporidians are a suitable model system for studying the spatio-temporal dynamics of pathogen emergence.

## Data Availability

The data presented in this study are available in Supplementary Materials: Tables S1 and S2.

## Supplementary Materials

The following supporting information can be downloaded at: https://www.mdpi.com/article/10.3390/microorganisms10051019/s1, Table S1: CCA data; Table S2: PCA data; Table S3: Results of CCA and references to haemoproteid data compiled from the MalAvi database; Table S4: Results of PCA and references to haemoproteid data compiled from the MalAvi database. References [3,5,21,24,25,26,33,56,65,69,70,93,94,95,96,97,98,99,100,101,102,103,104,105,106,107,108,109,110,111,112,113] are cited in the supplementary materials.
