# Supplementary material for: Phylogeographic Patterns of Haemoproteid Assemblages of Selected Avian Hosts: Ecological and Evolutionary Implications
Source: Microorganisms. 2022 May 12;10(5):1019. doi: 10.3390/microorganisms10051019 (PMC9144617; doi:10.3390/microorganisms10051019)
Supplement: Supplementary file 1 [file microorganisms-10-01019-s001.zip › Tables S3 and S4.pdf]

Table S3: Results of CCA analysis and data sources

Partitioning of scaled Chi-square:

	Inertia	Proportion
Total	11.1015	1.00000
Constrained	0.8353	0.07524
Unconstrained	10.2662	0.92476

Eigenvalues, and their contribution to the scaled Chi-square

Importance of components:

	CCA1	CCA2	CCA3	CA1	CA2	CA3	CA4	CA5	CA6	CA7
Eigenvalue	0.37468	0.23828	0.22231	1.00000	1.00000	0.98077	0.7427	0.60897	0.58447	0.5484
Proportion Explained	0.03375	0.02146	0.02003	0.09008	0.09008	0.08835	0.0669	0.05486	0.05265	0.0494
Cumulative Proportion	0.03375	0.05521	0.07524	0.16532	0.25540	0.34374	0.4106	0.46550	0.51815	0.5676

	CA8	CA9	CA10	CA11	CA12	CA13	CA14	CA15	CA16
Eigenvalue	0.51123	0.49266	0.44215	0.40383	0.33298	0.3053	0.28213	0.24750	0.22057
Proportion Explained	0.04605	0.04438	0.03983	0.03638	0.02999	0.0275	0.02541	0.02229	0.01987
Cumulative Proportion	0.61360	0.65798	0.69781	0.73418	0.76418	0.7917	0.81709	0.83938	0.85925

	CA17	CA18	CA19	CA20	CA21	CA22	CA23	CA24	CA25
Eigenvalue	0.21479	0.19567	0.18419	0.17134	0.12983	0.12158	0.110282	0.09159	0.084107
Proportion Explained	0.01935	0.01763	0.01659	0.01543	0.01169	0.01095	0.009934	0.00825	0.007576
Cumulative Proportion	0.87860	0.89623	0.91282	0.92825	0.93995	0.95090	0.960831	0.96908	0.976658

	CA26	CA27	CA28	CA29	CA30	CA31	CA32	CA33
Eigenvalue	0.062848	0.058074	0.037689	0.036325	0.022247	0.017082	0.01599	0.0083655
Proportion Explained	0.005661	0.005231	0.003395	0.003272	0.002004	0.001539	0.00144	0.0007536
Cumulative Proportion	0.982319	0.987550	0.990945	0.994217	0.996221	0.997760	0.99920	0.9999537

	CA34
Eigenvalue	5.137e-04
Proportion Explained	4.627e-05
Cumulative Proportion	1.000e+00

Accumulated constrained eigenvalues
Importance of components:

	CCA1	CCA2	CCA3
Eigenvalue	0.3747	0.2383	0.2223
Proportion Explained	0.4486	0.2853	0.2662
Cumulative Proportion	0.4486	0.7338	1.0000

Scaling 1 for species and site scores
* Sites are scaled proportional to eigenvalues
* Species are unscaled: weighted dispersion equal on all dimensions

Species scores

	CCA1	CCA2	CCA3	CA1	CA2	CA3
ARW1	-2.56248	-3.9410	-2.27766	5.844e-16	1.508e-15	-0.0969445
BLUTI09	1.01847	-1.8842	3.53795	1.624e-15	3.891e-15	-0.2351481
CCF1	0.25539	0.7516	0.09684	-9.426e-16	3.103e-15	0.1453400
CCF2	-1.24190	0.1054	0.02921	5.705e-16	3.932e-15	0.2338030
CF23	1.38599	0.5322	-1.64010	6.581e-16	1.995e-15	0.0030613
CCF3	0.74438	0.9055	-1.11574	4.815e-16	1.919e-15	0.0723253
CCF4	1.38599	0.5322	-1.64010	-1.661e-15	-2.370e-15	0.0149942
CCF5	1.38599	0.5322	-1.64010	7.973e-16	-2.606e-15	0.0101700
CCF6	-1.05594	0.4328	-0.24627	2.016e-17	1.448e-15	0.2309588
COLL2	1.38599	0.5322	-1.64010	-3.304e-15	-5.925e-16	0.0005217
CWT2	1.38599	0.5322	-1.64010	-1.546e-15	5.152e-15	-0.0029046
CWT4	1.20223	-0.6760	0.94893	-1.894e-16	1.352e-15	0.0032545
CYACAE08	1.38599	0.5322	-1.64010	-6.689e-16	-5.323e-15	0.0056009
CYACAE09	1.38599	0.5322	-1.64010	-1.162e-15	1.698e-16	0.0056009
GW3	1.01847	-1.8842	3.53795	-4.865e-16	-7.288e-15	-0.1054097
HIPOL1	-1.82206	2.3985	0.98169	2.024e+00	1.378e+01	-9.7257802
LK03	-2.56248	-3.9410	-2.27766	-7.694e-16	-6.783e-16	-0.2002849

PABY06	1.01847	-1.8842	3.53795	-3.534e-15	2.380e-15	0.0411538
PARUS1	0.12313	-0.7184	-0.21026	-8.258e-17	5.601e-16	0.0153291
PARUS10	1.38599	0.5322	-1.64010	2.796e-15	1.489e-15	-0.1194165
PARUS5	1.38599	0.5322	-1.64010	-1.302e-15	8.207e-17	-0.0029046
PARUS6	1.29411	-0.0719	-0.34559	-2.293e-16	1.210e-15	-0.0115362
PARUS65	1.38599	0.5322	-1.64010	-2.220e-16	1.977e-15	-0.0393520
PHSIB1	1.24817	-0.3739	0.30167	-4.292e-16	1.381e-15	-0.0221921
PHSIB2	1.38599	0.5322	-1.64010	-3.715e-15	5.682e-15	0.0056009
ROBIN1	1.38599	0.5322	-1.64010	4.171e-15	9.469e-15	0.0056009
ROFI1	1.38599	0.5322	-1.64010	4.540e-16	1.298e-15	-0.0244641
SFC1	1.01847	-1.8842	3.53795	7.766e-16	4.143e-16	-0.2351481
SYAT01	-0.83942	0.7422	0.29554	-3.749e-16	3.074e-15	0.2352604
SYAT02	0.34353	-0.1941	0.19083	-4.827e-17	8.514e-16	0.0719642
SYAT03	0.63413	0.1806	0.43768	1.651e-16	3.182e-15	0.0880814
SYAT04	-0.40179	0.2572	2.25982	3.761e-15	3.551e-15	0.1979875
SYAT07	-0.04139	0.9869	0.22325	2.360e-15	1.375e-15	0.1719038
SYAT09	-1.82206	2.3985	0.98169	-1.167e-14	3.649e-15	0.3548211
SYAT10	-0.56020	0.3277	0.60498	5.129e-16	2.343e-15	0.2308565
SYAT11	0.67088	0.4222	-0.08013	1.489e-17	1.486e-15	0.0798386
SYAT12	0.19414	0.3489	0.95985	8.649e-16	2.238e-15	0.1338586
SYAT13	0.55059	0.1415	0.66854	1.007e-15	1.337e-15	0.0990506
SYAT14	0.49210	0.3947	0.30986	2.617e-16	1.396e-15	0.1005244
SYAT16	0.49210	0.3947	0.30986	3.920e-16	2.077e-16	0.1074896
SYAT17	0.19414	0.3489	0.95985	8.649e-16	2.238e-15	0.1338586
SYAT18	-0.21803	1.4654	-0.32920	1.088e-15	7.291e-16	0.1776714
SYAT19	-1.82206	2.3985	0.98169	3.536e-15	3.649e-15	0.3548211
SYAT21	-0.21803	1.4654	-0.32920	-1.434e-15	1.744e-15	0.1832366
SYAT26	-1.82206	2.3985	0.98169	1.092e+01	-8.643e+00	-9.7257802
SYAT28	1.26349	-0.2733	0.08592	-2.306e-15	1.670e-16	0.0200589
SYAT29	1.01847	-1.8842	3.53795	8.360e-16	3.337e-15	0.0429236
SYAT30	1.01847	-1.8842	3.53795	-1.665e-15	3.719e-15	0.0411538
SYAT31	1.01847	-1.8842	3.53795	-1.665e-15	3.719e-15	0.0411538
SYAT33	1.20223	-0.6760	0.94893	-6.748e-16	1.405e-15	0.0233774
SYAT34	1.01847	-1.8842	3.53795	-1.665e-15	3.719e-15	0.0411538
SYAT35	1.14098	-1.0787	1.81194	1.335e-16	3.801e-15	0.0158876
SYAT36	-1.82206	2.3985	0.98169	-4.080e-16	6.499e-15	0.5533672
SYAT37	-1.82206	2.3985	0.98169	-2.378e-15	4.127e-15	0.4141076
SYAT41	-1.82206	2.3985	0.98169	-1.295e+01	-5.137e+00	-9.7257802
SYAT44	1.38599	0.5322	-1.64010	-5.048e-16	-1.565e-15	0.0030613
SYAT46	-1.82206	2.3985	0.98169	1.879e-15	8.373e-15	0.6926268
SYAT50	1.38599	0.5322	-1.64010	3.255e-16	-1.139e-15	0.0056009
SYAT51	1.38599	0.5322	-1.64010	3.255e-16	-1.139e-15	0.0056009
SYAT52	1.38599	0.5322	-1.64010	3.255e-16	-1.139e-15	0.0056009
SYBOR03	1.38599	0.5322	-1.64010	-1.272e-15	-1.636e-15	0.0005217
SYBOR04	1.38599	0.5322	-1.64010	-1.272e-15	-1.636e-15	0.0005217
SYBOR15	1.20223	-0.6760	0.94893	-1.564e-15	9.858e-16	0.0208378
SYBOR35	1.38599	0.5322	-1.64010	3.255e-16	-1.139e-15	0.0056009
TUCHR01	-2.56248	-3.9410	-2.27766	2.358e-16	-3.343e-16	-0.2002849
TUMER17	-2.56248	-3.9410	-2.27766	2.358e-16	-3.343e-16	-0.2002849
TURDUS2	-0.88598	-1.6874	-0.74158	-5.672e-16	-1.069e-16	0.0163089
TURMER08	1.38599	0.5322	-1.64010	3.255e-16	-1.139e-15	0.0056009
WW1	1.01847	-1.8842	3.53795	-1.030e-15	4.633e-16	-0.2351481
WW2	1.27574	-0.1927	-0.08668	-9.007e-16	1.148e-15	-0.0042881
WW5	1.38599	0.5322	-1.64010	-1.272e-15	-1.636e-15	0.0005217

Site scores (weighted averages of species scores)

	CCA1	CCA2	CCA3	CA1	CA2	CA3
row1	-0.78002	-0.22509	-0.17467	1.226e-16	6.416e-16	0.0637419
row2	-0.88598	-1.68743	-0.74158	-9.810e-16	1.990e-16	-0.0662813
row3	-0.88598	-1.68743	-0.74158	-3.711e-15	-2.899e-15	-0.0662813
row4	-0.88598	-1.68743	-0.74158	2.166e-15	3.039e-15	-0.0662813
row5	-0.88598	-1.68743	-0.74158	3.546e-16	1.279e-15	-0.0662813
row6	-0.88598	-1.68743	-0.74158	-3.090e-15	-2.475e-15	-0.0662813
row7	-0.76517	-0.46691	-0.29223	3.405e-16	1.094e-15	0.0415098
row8	-1.69006	-2.84574	-1.55696	2.393e-17	-1.792e-15	-0.1964335
row9	-1.14892	0.26910	-0.10853	3.291e-16	2.563e-15	0.1497908

row10	-0.46641	-0.14282	-0.22827	5.096e-15	7.010e-16	0.0405538
row11	0.31672	-0.20975	0.70140	-1.777e-17	7.349e-16	0.0420982
row12	0.15840	0.33054	-0.07871	-1.072e-14	-1.623e-16	0.0589271
row13	0.75456	-0.09311	-0.92518	1.444e-15	-4.167e-16	-0.1171202
row14	0.45626	-0.02869	0.20993	4.659e-15	7.956e-16	0.0134595
row15	0.57080	-1.30128	1.66384	2.292e-16	-5.335e-15	-0.1033827
row16	0.68565	-0.54617	0.04570	-1.210e-14	-1.040e-15	-0.0617739
row17	0.31297	-0.41446	0.28576	2.037e-15	-1.223e-15	0.0058721
row18	0.49201	-0.29559	1.31587	-2.915e-16	5.263e-16	0.0403624
row19	-0.88598	-1.68743	-0.74158	-5.950e-16	-1.200e-15	-0.0662813
row20	0.08441	0.08155	-0.43757	2.950e-15	1.111e-15	0.0322604
row21	-0.24876	0.50544	-0.54326	1.107e-14	1.931e-15	0.3400884
row22	-1.14892	0.26910	-0.10853	-1.669e-15	3.660e-15	0.4194052
row23	-1.14892	0.26910	-0.10853	-1.931e-15	3.842e-15	0.4194052
row24	-0.52096	0.56916	0.02576	2.125e-14	3.862e-15	0.3825256
row25	-1.14892	0.26910	-0.10853	-1.086e-15	4.352e-15	0.4194052
row26	-0.94768	0.58748	0.02463	-9.441e-16	0.000e+00	0.4201339
row27	-0.40028	0.59217	-0.07472	-1.961e-14	3.013e-15	0.3751736
row28	-1.04575	0.42680	0.02616	-1.804e-15	3.912e-15	0.4203650
row29	-1.05594	0.43277	-0.24627	6.442e-15	4.250e-15	0.4179831
row30	-0.88598	-1.68743	-0.74158	-3.803e-15	1.834e-15	0.2033332
row31	-0.69981	0.53494	0.45026	8.542e-16	4.541e-15	0.4200827
row32	-1.14892	0.26910	-0.10853	-1.940e-15	3.860e-15	0.4194052
row33	-1.14892	0.26910	-0.10853	-1.901e-15	3.823e-15	0.4194052
row34	0.12313	-0.71840	-0.21026	-3.181e-16	-1.864e-15	-0.0497473
row35	-0.83942	0.74220	0.29554	4.642e-16	4.063e-15	0.4222847
row36	-0.83942	0.74220	0.29554	-1.852e-15	4.063e-15	0.4222847
row37	-0.94768	0.58748	0.02463	2.141e-15	4.270e-15	0.4201339
row38	-0.83942	0.74220	0.29554	-1.852e-15	4.063e-15	0.4222847
row39	-1.00581	-0.10176	-0.16578	-2.332e-15	3.507e-15	0.3661070
row40	-0.83942	0.74220	0.29554	-1.852e-15	4.063e-15	0.4222847
row41	-0.55939	-0.30648	-0.09052	-5.306e-15	2.676e-15	0.3115903
row42	-0.97096	-0.62733	-0.49393	3.388e-15	1.189e-15	0.0652914
row43	-0.86270	-0.47262	-0.22302	-2.777e-15	2.757e-15	0.3128089
row44	-1.24190	0.10544	0.02921	-7.825e-15	2.240e-15	0.1754606
row45	-0.83942	0.74220	0.29554	-1.852e-15	4.063e-15	0.4222847
row46	-0.88598	-1.68743	-0.74158	-1.543e-15	2.929e-16	-0.0420335
row47	-0.71104	-0.38955	-0.15677	-1.912e-15	3.603e-16	0.0425852
row48	0.56702	0.20935	-0.52484	-2.045e-15	3.875e-16	-0.0028488
row49	-1.10947	-2.06754	-1.04341	2.050e-16	-2.321e-15	-0.0950803
row50	-1.24190	0.10544	0.02921	-7.323e-15	1.887e-15	0.1512129
row51	-0.83538	-0.04619	-0.02183	3.126e-15	1.258e-15	0.0957560
row52	0.62672	0.26146	-0.48325	7.253e-17	-4.865e-16	0.0054932
row53	-1.82206	2.39852	0.98169	2.024e+00	1.378e+01	-9.5387559
row54	-0.34053	0.81960	0.52156	-2.977e-15	5.761e-15	0.4061444
row55	-1.82206	2.39852	0.98169	-1.295e+01	-5.137e+00	-9.5387559
row56	-0.83942	0.74220	0.29554	-1.852e-15	4.063e-15	0.4222847
row57	-1.82206	2.39852	0.98169	1.092e+01	-8.643e+00	-9.5387559
row58	0.12313	-0.71840	-0.21026	-3.972e-16	1.883e-15	0.2023534
row59	-0.13107	0.66826	0.48439	5.907e-17	3.483e-15	0.3479980
row60	0.12313	-0.71840	-0.21026	-1.755e-16	-1.864e-15	-0.0497473
row61	0.62261	0.20553	-0.34640	-9.969e-16	-1.476e-16	0.0005117
row62	0.26881	-0.17415	0.02639	1.173e-14	6.937e-16	0.0068172
row63	0.75456	-0.09311	-0.92518	2.833e-15	-3.891e-15	-0.0696440
row64	1.00826	-0.18822	-0.40884	1.906e-15	-1.370e-15	-0.0777022
row65	0.34445	0.02739	-0.08424	-4.793e-15	3.699e-16	0.0114280
row66	0.98529	-0.33924	-0.08522	-4.183e-15	-8.291e-16	-0.0640142
row67	-1.40144	1.70825	0.85612	1.472e-15	5.848e-15	0.6793077
row68	0.54236	-1.83497	2.46807	-5.706e-17	-1.723e-15	-0.2306263

Site constraints (linear combinations of constraining variables)

	CCA1	CCA2	CCA3	CA1	CA2	CA3
row1	-0.9601	-0.9390	-0.5063	1.226e-16	6.416e-16	0.0637419
row2	-0.9601	-0.9390	-0.5063	-9.810e-16	1.990e-16	-0.0662813
row3	-0.9601	-0.9390	-0.5063	-3.711e-15	-2.899e-15	-0.0662813
row4	-0.9601	-0.9390	-0.5063	2.166e-15	3.039e-15	-0.0662813

row5	-0.9601	-0.9390	-0.5063	3.546e-16	1.279e-15	-0.0662813
row6	-0.9601	-0.9390	-0.5063	-3.090e-15	-2.475e-15	-0.0662813
row7	-0.9601	-0.9390	-0.5063	3.405e-16	1.094e-15	0.0415098
row8	-0.9601	-0.9390	-0.5063	2.393e-17	-1.792e-15	-0.1964335
row9	-0.9601	-0.9390	-0.5063	3.291e-16	2.563e-15	0.1497908
row10	-0.9601	-0.9390	-0.5063	5.096e-15	7.010e-16	0.0405538
row11	0.3816	-0.4490	0.7865	-1.777e-17	7.349e-16	0.0420982
row12	0.5193	0.1268	-0.3646	-1.072e-14	-1.623e-16	0.0589271
row13	0.5193	0.1268	-0.3646	1.444e-15	-4.167e-16	-0.1171202
row14	0.3816	-0.4490	0.7865	4.659e-15	7.956e-16	0.0134595
row15	0.3816	-0.4490	0.7865	2.292e-16	-5.335e-15	-0.1033827
row16	0.3816	-0.4490	0.7865	-1.210e-14	-1.040e-15	-0.0617739
row17	0.3816	-0.4490	0.7865	2.037e-15	-1.223e-15	0.0058721
row18	0.3816	-0.4490	0.7865	-2.915e-16	5.263e-16	0.0403624
row19	-0.9601	-0.9390	-0.5063	-5.950e-16	-1.200e-15	-0.0662813
row20	0.5193	0.1268	-0.3646	2.950e-15	1.111e-15	0.0322604
row21	-0.6827	0.5715	0.2182	1.107e-14	1.931e-15	0.3400884
row22	-0.6827	0.5715	0.2182	-1.669e-15	3.660e-15	0.4194052
row23	-0.6827	0.5715	0.2182	-1.931e-15	3.842e-15	0.4194052
row24	-0.6827	0.5715	0.2182	2.125e-14	3.862e-15	0.3825256
row25	-0.6827	0.5715	0.2182	-1.086e-15	4.352e-15	0.4194052
row26	-0.6827	0.5715	0.2182	-9.441e-16	0.000e+00	0.4201339
row27	-0.6827	0.5715	0.2182	-1.961e-14	3.013e-15	0.3751736
row28	-0.6827	0.5715	0.2182	-1.804e-15	3.912e-15	0.4203650
row29	-0.6827	0.5715	0.2182	6.442e-15	4.250e-15	0.4179831
row30	-0.6827	0.5715	0.2182	-3.803e-15	1.834e-15	0.2033332
row31	-0.6827	0.5715	0.2182	8.542e-16	4.541e-15	0.4200827
row32	-0.6827	0.5715	0.2182	-1.940e-15	3.860e-15	0.4194052
row33	-0.6827	0.5715	0.2182	-1.901e-15	3.823e-15	0.4194052
row34	0.5193	0.1268	-0.3646	-3.181e-16	-1.864e-15	-0.0497473
row35	-0.6827	0.5715	0.2182	4.642e-16	4.063e-15	0.4222847
row36	-0.6827	0.5715	0.2182	-1.852e-15	4.063e-15	0.4222847
row37	-0.6827	0.5715	0.2182	2.141e-15	4.270e-15	0.4201339
row38	-0.6827	0.5715	0.2182	-1.852e-15	4.063e-15	0.4222847
row39	-0.6827	0.5715	0.2182	-2.332e-15	3.507e-15	0.3661070
row40	-0.6827	0.5715	0.2182	-1.852e-15	4.063e-15	0.4222847
row41	-0.6827	0.5715	0.2182	-5.306e-15	2.676e-15	0.3115903
row42	0.3816	-0.4490	0.7865	3.388e-15	1.189e-15	0.0652914
row43	-0.6827	0.5715	0.2182	-2.777e-15	2.757e-15	0.3128089
row44	0.3816	-0.4490	0.7865	-7.825e-15	2.240e-15	0.1754606
row45	-0.6827	0.5715	0.2182	-1.852e-15	4.063e-15	0.4222847
row46	0.3816	-0.4490	0.7865	-1.543e-15	2.929e-16	-0.0420335
row47	-0.9601	-0.9390	-0.5063	-1.912e-15	3.603e-16	0.0425852
row48	0.5193	0.1268	-0.3646	-2.045e-15	3.875e-16	-0.0028488
row49	-0.9601	-0.9390	-0.5063	2.050e-16	-2.321e-15	-0.0950803
row50	-0.9601	-0.9390	-0.5063	-7.323e-15	1.887e-15	0.1512129
row51	-0.9601	-0.9390	-0.5063	3.126e-15	1.258e-15	0.0957560
row52	0.5193	0.1268	-0.3646	7.253e-17	-4.865e-16	0.0054932
row53	-0.6827	0.5715	0.2182	2.024e+00	1.378e+01	-9.5387559
row54	-0.6827	0.5715	0.2182	-2.977e-15	5.761e-15	0.4061444
row55	-0.6827	0.5715	0.2182	-1.295e+01	-5.137e+00	-9.5387559
row56	-0.6827	0.5715	0.2182	-1.852e-15	4.063e-15	0.4222847
row57	-0.6827	0.5715	0.2182	1.092e+01	-8.643e+00	-9.5387559
row58	-0.6827	0.5715	0.2182	-3.972e-16	1.883e-15	0.2023534
row59	-0.6827	0.5715	0.2182	5.907e-17	3.483e-15	0.3479980
row60	0.5193	0.1268	-0.3646	-1.755e-16	-1.864e-15	-0.0497473
row61	0.5193	0.1268	-0.3646	-9.969e-16	-1.476e-16	0.0005117
row62	0.5193	0.1268	-0.3646	1.173e-14	6.937e-16	0.0068172
row63	0.5193	0.1268	-0.3646	2.833e-15	-3.891e-15	-0.0696440
row64	0.5193	0.1268	-0.3646	1.906e-15	-1.370e-15	-0.0777022
row65	0.5193	0.1268	-0.3646	-4.793e-15	3.699e-16	0.0114280
row66	0.3816	-0.4490	0.7865	-4.183e-15	-8.291e-16	-0.0640142
row67	-0.6827	0.5715	0.2182	1.472e-15	5.848e-15	0.6793077
row68	0.3816	-0.4490	0.7865	-5.706e-17	-1.723e-15	-0.2306263

Biplot scores for constraining variables

	CCA1	CCA2	CCA3	CA1	CA2	CA3
Mediterranean	-0.4059	0.3398	0.1298	0	0	0
temperate-oceanic	0.1904	-0.2240	0.3924	0	0	0
warm-temperate	-0.3550	-0.3472	-0.1872	0	0	0

Centroids for factor constraints

	CCA1	CCA2	CCA3	CA1	CA2	CA3
humid-continental	0.5193	0.1268	-0.3646	0	0	0
Mediterranean	-0.6827	0.5715	0.2182	0	0	0
temperate-oceanic	0.3816	-0.4490	0.7865	0	0	0
warm-temperate	-0.9601	-0.9390	-0.5063	0	0	0

Data sources from the MalAvi database

[3] [5] [21] [24-26] [33] [56] [65] [69-70] [93-112]

3. Bensch, S.; Stjernman, M.; Hasselquist, D.; Ostman, O.; Hansson, B.; Westerdahl, H.; Pinheiro, R.T. Host specificity in avian blood parasites: A study of Plasmodium and Haemoproteus mitochondrial DNA amplified from birds. Proceedings of the Royal Society B: Biological Sciences 2000, 267, 1583-1589, doi:10.1098/rspb.2000.1181.
5. Ellis, V.A.; Huang, X.; Westerdahl, H.; Jönsson, J.; Hasselquist, D.; Neto, J.M.; Nilsson, J.Å.; Nilsson, J.; Hegemann, A.; Hellgren, O.; et al. Explaining prevalence, diversity and host specificity in a community of avian haemosporidian parasites. Oikos 2020, doi:10.1111/oik.07280.
21. Cosgrove, C.L.; Wood, M.J.; Day, K.P.; Sheldon, B.C. Seasonal variation in Plasmodium prevalence in a population of blue tits Cyanistes caeruleus. Journal of Animal Ecology 2008, 77, 540-548, doi:10.1111/j.1365-2656.2008.01370.x.
24. Palinauskas, V.; Iezhova, T.A.; Krizanauskiene, A.; Markovets, M.Y.; Bensch, S.; Valkiūnas, G. Molecular characterization and distribution of Haemoproteus minutus (Haemosporida, Haemoproteidae): A pathogenic avian parasite. Parasitology International 2013, 62, 358-363, doi:10.1016/j.parint.2013.03.006.
25. Podmokla, E.; Dubiec, A.; Arct, A.; Drobniak, S.M.; Gustafsson, L.; Cichoń, M. Malaria infection status predicts extra-pair paternity in the blue tit. Journal of Avian Biology 2015, 46, 303-306, doi:10.1111/jav.00599.
26. Nilsson, E.; Taubert, H.; Hellgren, O.; Huang, X.; Palinauskas, V.; Markovets, M.Y.; Valkiūnas, G.; Bensch, S. Multiple cryptic species of sympatric generalists within the avian blood parasite Haemoproteus majoris. Journal of evolutionary biology 2016, 29, 1812-1826, doi:10.1111/jeb.12911.
33. Šujanová, A.; Špitalská, E.; Václav, R. Seasonal Dynamics and Diversity of Haemosporidians in a Natural Woodland Bird Community in Slovakia. Diversity 2021, 13, 439.
56. Križanauskiene, A.; Hellgren, O.; Kosarev, V.; Sokolov, L.; Bensch, S.; Valkiūnas, G. Variation in host specificity between species of avian hemosporidian parasites: Evidence from parasite morphology and cytochrome B gene sequences. Journal of Parasitology 2006, 92, 1319-1324, doi:10.1645/GE-873R.1.
65. Ilgūnas, M.; Romeiro Fernandes Chagas, C.; Bukauskaitė, D.; Bernotienė, R.; Iezhova, T.; Valkiūnas, G. The life-cycle of the avian haemosporidian parasite Haemoproteus majoris, with emphasis on the exoerythrocytic and sporogonic development. Parasites and Vectors 2019, 12, doi:10.1186/s13071-019-3773-4.
69. Santiago-Alarcon, D.; Bloch, R.; Rolshausen, G.; Schaefer, H.M.; Segelbacher, G. Prevalence, diversity, and interaction patterns of avian haemosporidians in a four-year study of blackcaps in a migratory divide. Parasitology 2011, 138, 824-835, doi:10.1017/s0031182011000515.
70. Drovetski, S.V.; Aghayan, S.A.; Mata, V.A.; Lopes, R.J.; Mode, N.A.; Harvey, J.A.; Voelker, G. Does the niche breadth or trade-off hypothesis explain the abundance-occupancy relationship in avian Haemosporidia? Molecular Ecology 2014, 23, 3322-3329, doi:10.1111/mec.12744.
93. Hellgren, O.; Krizanauskiene, A.; Valkiūnas, G.; Bensch, S. Diversity and phylogeny of mitochondrial cytochrome B lineages from six morphospecies of avian Haemoproteus (Haemosporida : Haemoproteidae). Journal of Parasitology 2007, 93, 889-896, doi:10.1645/ge-1051r1.1.
94. Dimitrov, D.; Zehtindjiev, P.; Bensch, S. Genetic diversity of avian blood parasites in SE Europe: Cytochrome b lineages of the genera Plasmodium and Haemoproteus (Haemosporida) from Bulgaria. Acta Parasitologica 2010, 55, 201-209, doi:10.2478/s11686-010-0029-z.
95. Santiago-Alarcon, D.; MacGregor-Fors, I.; Kühnert, K.; Segelbacher, G.; Schaefer, H.M. Avian haemosporidian parasites in an urban forest and their relationship to bird size and abundance. Urban Ecosystems 2016, 19, 331-346, doi:10.1007/s11252-015-0494-0.

96. Bodawatta, K.H.; Synek, P.; Bos, N.; Garcia-del-Rey, E.; Koane, B.; Marki, P.Z.; Albrecht, T.; Lifjeld, J.; Poulsen, M.; Munclinger, P. Spatiotemporal patterns of avian host–parasite interactions in the face of biogeographical range expansions. Molecular ecology 2020, 29, 2431-2448.
97. Stanković, D.; Jönsson, J.; Raković, M. Diversity of avian blood parasites in wild passerines in Serbia with special reference to two new lineages. Journal of Ornithology 2019, 160, 545-555, doi:10.1007/s10336-019-01628-z.
98. Valkiūnas, G.; Iezhova, T.A.; Križanauskiene, A.; Palinauskas, V.; Sehgal, R.N.M.; Bensch, S. A comparative analysis of microscopy and PCR-based detection methods for blood parasites. Journal of Parasitology 2008, 94, 1395-1401, doi:10.1645/GE-1570.1.
99. Nourani, L.; Aliabadian, M.; Dinparast Djadid, N.; Mirshamsi, O. Occurrence of Haemoproteus spp. (Haemosporida: Haemoproteidae) in New Host Records of Passerine Birds from the East of Iran. Iranian Journal of Parasitology 2018, 13, 267-274.
100. Knowles, S.C.L.; Wood, M.J.; Alves, R.; Wilkin, T.A.; Bensch, S.; Sheldon, B.C. Molecular epidemiology of malaria prevalence and parasitaemia in a wild bird population. Molecular Ecology 2011, 20, 1062-1076, doi:10.1111/j.1365-294X.2010.04909.x.
101. Stjernman, M.; Råberg, L.; Nilsson, J.Å. Long-term effects of nestling condition on blood parasite resistance in blue tits (Cyanistes caeruleus). Canadian Journal of Zoology 2008, 86, 937-946, doi:10.1139/Z08-071.
102. Dubiec, A.; Podmokła, E.; Zagalska-Neubauer, M.; Drobniak, S.M.; Arct, A.; Gustafsson, L.; Cichoń, M. Differential prevalence and diversity of haemosporidian parasites in two sympatric closely related non-migratory passerines. Parasitology 2016, 143, 1320-1329, doi:10.1017/S0031182016000779.
103. Schumm, Y.R.; Wecker, C.; Marek, C.; Wassmuth, M.; Bentele, A.; Willems, H.; Reiner, G.; Quillfeldt, P. Blood parasites in Passeriformes in central Germany: prevalence and lineage diversity of Haemosporida (Haemoproteus, Plasmodium and Leucocytozoon) in six common songbirds. Peerj 2019, 6, doi:10.7717/peerj.6259.
104. Lynton-Jenkins, J.G.; Bründl, A.C.; Cauchoix, M.; Lejeune, L.A.; Sallé, L.; Thiney, A.C.; Russell, A.F.; Chaine, A.S.; Bonneaud, C. Contrasting the seasonal and elevational prevalence of generalist avian haemosporidia in co-occurring host species. Ecology and Evolution 2020, doi:10.1002/ece3.6355.
105. Wood, M.J.; Cosgrove, C.L.; Wilkin, T.A.; Knowles, S.C.L.; Day, K.P.; Sheldon, B.C. Within-population variation in prevalence and lineage distribution of avian malaria in blue tits, Cyanistes caeruleus. Molecular Ecology 2007, 16, 3263-3273, doi:10.1111/j.1365-294X.2007.03362.x.
106. Wiersch, S.C.; Lubjuhn, T.; Maier, W.A.; Kampen, H. Haemosporidian infection in passerine birds from Lower Saxony. Journal of Ornithology 2007, 148, 17-24, doi:10.1007/s10336-006-0094-0.
107. Garcia-Longoria, L.; Marzal, A.; De Lope, F.; Garamszegi, L. Host-parasite interaction explains variation in the prevalence of avian haemosporidians at the community level. PLoS ONE 2019, 14, doi:10.1371/journal.pone.0205624.
108. Glaizot, O.; Fumagalli, L.; Iritano, K.; Lalubin, F.; van Rooyen, J.; Christe, P. High prevalence and lineage diversity of avian malaria in wild populations of great tits (parus major) and mosquitoes (culex pipiens). PLoS ONE 2012, 7, doi:10.1371/journal.pone.0034964.
109. van Rooyen, J.; Lalubin, F.; Glaizot, O.; Christe, P. Avian haemosporidian persistence and co-infection in great tits at the individual level. Malaria Journal 2013, 12, doi:10.1186/1475-2875-12-40.
110. Pérez-Rodríguez, A.; Ramírez, A.; Richardson, D.S.; Pérez-Tris, J. Evolution of parasite island syndromes without long-term host population isolation: Parasite dynamics in Macaronesian blackcaps Sylvia atricapilla. Global Ecology and Biogeography 2013, 22, 1272-1281, doi:10.1111/geb.12084.
111. Valkiūnas, G.; Iezhova, T.A.; Evans, E.; Carlson, J.S.; Martínez-Gómez, J.E.; Sehgal, R.N.M. Two new Haemoproteus species (Haemosporida: Haemoproteidae) from columbiform birds. Journal of Parasitology 2013, 99, 513-521, doi:10.1645/12-98.1.
112. Martínez, J.; Vásquez, R.A.; Venegas, C.; Merino, S. Molecular characterisation of haemoparasites in forest birds from Robinson Crusoe Island: Is the Austral Thrush a potential threat to endemic birds? Bird Conservation International 2015, 25, 139-152, doi:10.1017/S0959270914000227.

Table S4: Results of PCA analysis and data sources

Partitioning of correlations:

	Inertia	Proportion
Total	39	1
Unconstrained	39	1

Eigenvalues, and their contribution to the correlations

Importance of components:

	PC1	PC2	PC3	PC4	PC5
Eigenvalue	11.5287	11.0491	7.7840	6.5945	2.0436
Proportion Explained	0.2956	0.2833	0.1996	0.1691	0.0524
Cumulative Proportion	0.2956	0.5789	0.7785	0.9476	1.0000

Scaling 1 for species and site scores
* Sites are scaled proportional to eigenvalues
* Species are unscaled: weighted dispersion equal on all dimensions
* General scaling constant of scores: 3.736876

Species scores

	PC1	PC2	PC3	PC4	PC5
CCF2	1.0067	0.02433	0.27357	-0.45466	0.4009
CWT2	0.1281	-0.15184	-0.04953	1.42739	0.1785
CWT4	1.0067	0.02433	0.27357	-0.45466	0.4009
PABY06	-0.4524	0.66560	0.92419	-0.07352	0.1108
PARUS1	-0.1314	-0.74812	0.13044	1.04618	0.3363
ROBIN1	-0.3938	-0.96683	0.27858	-0.40648	0.2750
SYAT01	-0.7769	0.55975	-0.20167	-0.67891	-0.3034
SYAT02	-0.4471	0.01092	0.40814	1.20107	-0.6443
SYAT03	0.5853	-0.83925	0.43026	0.08305	-0.6044
SYAT04	-0.4524	0.66560	0.92419	-0.07352	0.1108
SYAT07	-0.6229	-0.09055	-1.01024	-0.34289	0.5774
SYAT10	-0.3328	0.47442	-0.56317	-1.08055	0.1248
SYAT11	0.3219	-0.43588	0.38603	1.16578	0.5181
SYAT12	-0.6015	-0.62962	0.71358	-0.43321	0.3222
SYAT13	-0.2194	-0.10405	-0.71344	-1.13454	-0.6409
SYAT14	0.4731	-0.03805	0.02878	-0.67787	-2.0190
SYAT16	0.3438	-0.99856	0.19104	0.42972	0.2008
SYAT17	-0.6304	0.19360	1.03813	-0.26497	0.2392
SYAT18	1.0067	0.02433	0.27357	-0.45466	0.4009
SYAT28	-0.3938	-0.96683	0.27858	-0.40648	0.2750
SYAT30	-0.4524	0.66560	0.92419	-0.07352	0.1108
SYAT31	-0.4524	0.66560	0.92419	-0.07352	0.1108
SYAT32	-0.4524	0.66560	0.92419	-0.07352	0.1108
SYAT33	-0.6442	-0.46453	0.83564	-0.41751	0.3209
SYAT34	-0.4524	0.66560	0.92419	-0.07352	0.1108
SYAT35	0.6456	0.43397	0.82299	-0.46339	0.4370
SYAT36	-0.2498	0.32222	-0.96425	-0.29570	1.4478
SYAT37	-0.2498	0.32222	-0.96425	-0.29570	1.4478
SYAT44	0.3881	-0.71457	0.13124	-0.79934	-1.0467
SYAT50	-0.3938	-0.96683	0.27858	-0.40648	0.2750
SYAT51	-0.3938	-0.96683	0.27858	-0.40648	0.2750
SYAT52	-0.3938	-0.96683	0.27858	-0.40648	0.2750
SYBOR03	1.0067	0.02433	0.27357	-0.45466	0.4009
SYBOR04	1.0067	0.02433	0.27357	-0.45466	0.4009
SYBOR15	0.7776	0.33498	0.70071	-0.48105	0.4456
SYBOR35	-0.3938	-0.96683	0.27858	-0.40648	0.2750
TURDUS2	-0.3938	-0.96683	0.27858	-0.40648	0.2750
WW2	0.9628	-0.24000	0.26830	0.47423	0.5412
WW5	1.0067	0.02433	0.27357	-0.45466	0.4009

Site scores (weighted sums of species scores)

	PC1	PC2	PC3	PC4	PC5
Germany	-0.76247	1.07503	1.05157	-0.07087	0.03311

Portugal	-0.06539	0.17204	-0.52631	-0.18994	-0.72086
Russia	0.21591	-0.24524	-0.05636	1.37596	0.05333
Slovakia	-0.66362	-1.56154	0.31698	-0.39183	0.08216
Spain	-0.42092	0.52043	-1.09716	-0.28504	0.43250
Sweden	1.69650	0.03929	0.31128	-0.43828	0.11975

Data sources from the MalAvi database

Sweden [5], Slovakia [33], Russia [56], Spain [58], Germany [69], and Portugal [113].

5. Ellis, V.A.; Huang, X.; Westerdahl, H.; Jönsson, J.; Hasselquist, D.; Neto, J.M.; Nilsson, J.-A.; Nilsson, J.; Hegemann, A.; Hellgren, O. Explaining Prevalence, Diversity and Host Specificity in a Community of Avian Haemosporidian Parasites. *Oikos* **2020**, *129*, 1314–1329.
33. Šujanová, A.; Špitalská, E.; Václav, R. Seasonal Dynamics and Diversity of Haemosporidians in a Natural Woodland Bird Community in Slovakia. *Diversity* **2021**, *13*, 439.
56. Križanauskienė, A.; Hellgren, O.; Kosarev, V.; Sokolov, L.; Bensch, S.; Valkiūnas, G. Variation in Host Specificity between Species of Avian Hemosporidian Parasites: Evidence from Parasite Morphology and Cytochrome b Gene Sequences. *Journal of Parasitology* **2006**, *92*, 1319–1324, doi:10.1645/GE-873R.1.
58. Pérez-Rodríguez, A.; de la Hera, I.; Bensch, S.; Pérez-Tris, J. Evolution of Seasonal Transmission Patterns in Avian Blood-Borne Parasites. *International Journal for Parasitology* **2015**, *45*, 605–611, doi:10.1016/j.ijpara.2015.03.008.
69. Santiago-Alarcon, D.; Bloch, R.; Rolshausen, G.; Schaefer, H.M.; Segelbacher, G. Prevalence, Diversity, and Interaction Patterns of Avian Haemosporidians in a Four-Year Study of Blackcaps in a Migratory Divide. *Parasitology* **2011**, *138*, 824–835.
113. Mata, V.A.; da Silva, L.P.; Lopes, R.J.; Drovetski, S.V. The Strait of Gibraltar Poses an Effective Barrier to Host-Specialised but Not to Host-Generalised Lineages of Avian Haemosporidia. *International Journal for Parasitology* **2015**, *45*, 711–719, doi:10.1016/j.ijpara.2015.04.006.
